# Substantive molecular and histological changes within the meniscus with tears

**DOI:** 10.1186/s12891-019-2943-z

**Published:** 2019-12-01

**Authors:** Yi Long, Jingping Xie, Zhi-Qi Zhang, Ziji Zhang, Fangang Meng, Aishan He

**Affiliations:** 1 Department of Joint Surgery, First Affiliated Hospital of Sun Yat-sen University, Guangzhou, 510080 Guangdong China; 2 Department of Orthopedics, The Central Hospital of Shao Yang, Shaoyang, 422000 Hunan China

**Keywords:** Meniscal tear, Anterior cruciate ligament tear, Gene, MicroRNA, Meniscus degeneration

## Abstract

**Background:**

The meniscus plays a vital role in the normal biomechanics of the knee. However, it is not well studied at the molecular level. The purpose of this study was to determine whether molecular and pathological changes in the meniscal tissue vary depending on the presence or absence of meniscal and/or anterior cruciate ligament tear (ACL).

**Methods:**

Six normal menisci (group A), seven simple torn menisci (group B) and seven torn menisci with concomitant anterior cruciate ligament tears (group C) were collected. We observed the pathological changes in the menisci and used real-time polymerase chain reaction along with immunohistochemistry and in situ hybridisation to examine the levels of *ACAN, ADAMTS5, COL10A1, CEBPβ, MMP13* and *miR-381-3p, miR-455-3p, miR-193b-3p, miR-92a-3p,* respectively. Patients were scored preoperatively and postoperatively using the Lysholm Knee Scoring Scale and International Knee Documentation Committee Subjective Knee Evaluation Form.

**Results:**

Compared with group A, the expression levels of *ADAMTS5, COL10A1, CEBPβ,* and *MMP13* and all the miRNAs were increased while *ACAN* was down-regulated in groups B and C. Additionally, the gene expression and miRNA levels were higher in group C than that in group B, except for *ACAN*, which was lower. Several fibrochondrocytes strongly expressed ADAMTS5, CEBPβ, and MMP13 in groups B and C and had high levels of *miR-381-3p* and *miR-455-3p* than that in group A. Postoperative Lysholm and IKDC scores were higher in group B than in group C.

**Conclusions:**

Our findings suggest that the meniscus tended to degenerate after it was injured, especially when combined with a torn ACL. The miRNAs investigated in this study might also contribute to meniscus degeneration. Patients with a combined injury patterns might have relatively worse joint function.

## Background

It is well known that meniscus plays a critical role in the normal biomechanics of the tibiofemoral joint, and meniscal damaged by trauma dramatically increases the risk of osteoarthritis (OA) during middle and old age [1]. An important reason behind the acceleration of joint degeneration, including the cartilage and meniscus, is probably due to disruption of the knee-joint’s biomechanics following meniscus injury. Moreover, the underlying molecular mechanism and gene expression changes might be other significant causes [2]. Previous studies on the meniscus have primarily focused on the biomechanics [3–7], and only a few investigations have studied its molecular aspects [8, 9].

The meniscus comprises dense fibrocartilage that is populated with cells known as fibrochondrocytes. These cells synthesise and maintain the extracellular matrix (ECM), which is primarily composed of type I collagen (COL1A1) and other components such as aggrecan (ACAN), elastin, as well as small amounts of other types of collagen [1, 10]. In physiological situations, there is a dynamic balance between ECM synthesis and degradation [11]. The roles of matrix metalloproteinases 13 (MMP13) and an ‘aggrecanase’ known as a disintegrin and metalloproteinase with thrombospondin motifs − 5 (ADAMTS5), in OA through the degradation of ECM are well established [12–14]. It is also clear that type X collagen (COL10A1) is a marker of fibrochondrocyte hypertrophy in meniscus [9, 15], and CEBPβ contributes to the pathophysiological process of OA [16, 17]. These genes whose expression level corretated with the extent of cartilage degeneration might be considered as markers of articular deterioration.

Recent advances in epigenetic research have shed light on the importance of microRNA (miRNA) in the regulation of gene expression at multiple levels related to the pathogenesis of OA [18]. In a previous study, we reported significant up-regulation of the miRNAs, miR-381-3p, miR-455-3p, miR-193b-3p, and miR-92a-3p during differentiation of human mesenchymal stem cells into chondrocytes, and provided evidence that these four miRNAs may regulate early chondrogenesis and cartilage degeneration [19–24]. However, the expression profile of the miRNAs in the menisci with tears is unknown. The purpose of this research was to determine the changes in gene expression in the meniscus depending on whether the meniscal tear is accompanied by ACL tear or not, suggeting that the meniscal tissue tends to degenerate at the molecular level after injury, especially when combined with a torn ACL.

## Methods

### Tissue acquisition and processing

Six normal meniscal tissues without tears were collected from amputees suffering from osteosarcoma simultaneously with the samples for the control group (group A; four males and two females; age range: 16–26 years; body mass index (BMI) range: 18.5–22.5 kg/m^2^). Additionally, meniscal tissues (experimental group) were collected from 14 patients undergoing partial meniscectomy, including seven patients with simple meniscal tears (group B; three males and four females; age range: 16–38 years; BMI range: 19.6–25.3 kg/m^2^) and seven patients with meniscal and concomitant ACL tears (group C; four males and three females; age range: 17–34 years; BMI range: 20.6–26.7 kg/m^2^). All arthroscopic surgeries were performed by one of the authors, and the level of cartilage injury in all of the patients was ≤ Grade II (Outerbridge Classification System) [25]. For meniscectomy patients, medial or lateral menisci from 14 knees were obtained from the middle portion or/and posterior horn. All types of tears were included, such as radial, flap, and complex. The demographic characteristics of these patients are presented in Table 1. None of the patients had advanced gouty arthritis, rheumatoid arthritis, osteoarthritis, or any additional posterior cruciate or collateral ligament injury at the time of the meniscal surgery. All meniscal tissues were harvested from the inner zone. The labelled specimens were transported to the laboratory from the operating room in sterile screw-cap containers.

**Table 1:** Demographic Data

	Group A (normal meniscus)	Group B (meniscal tear)	Group C (meniscal tear and ACL tear)
All patients			
Males	4	3	4
Females	2	4	3
Total	6	7	7
Mean age (range) *(years)*	21.5 (16–26)	22.4 (16–38)	27.4 (17–34)
BMI (range) *(kg/m*^*2*^*)*	20.8 (18.5–22.5)	22.7 (19.6–25.3)	23.3 (20.6–26.7)
Time interval from injury to surgery *(months)*	NA	12.3 (3–24)	11.7 (3–18)
Outerbridge classification system			
Grade 0	4	1	1
Grade I	2	5	3
Grade II	0	1	3
Type of meniscal tear			
Radial	NA	2	3
Flap	NA	3	1
Longitudinal and flap	NA	1	2
Horizontal and flap	NA	1	1
Location of meniscal tear			
Medial posterior horn	NA	3	2
Lateral posterior horn	NA	1	2
Medial middle portion	NA	0	2
Lateral middle portion	NA	1	0
Medial posterior horn and middle portion	NA	2	1

ACL: anterior cruciate ligament. BMI: body mass index

### Total RNA extraction and quantitative real-time PCR analysis

Total RNA was extracted with the miRNeasy Mini Kit (QIAGEN, Germany) according to the manufacturer’s instructions. The yield and quality were spectrophotometrically assessed using the Epoch spectrophotometer (Biotek, USA). The cDNA from the mRNAs and miRNAs was obtained using Prime-Script RT Master Mix (Takara, Japan) and PrimeScript miRNA cDNA Synthesis Kit (Takara, Japan), respectively, following the manufacturer’s instructions. The mRNAs quantitative real-time PCR (qRT-PCR) was performed with THUNDERBIRD SYBR qPCR Mix (TOYOBO, Japan) while the miRNAs qRT-PCR was performed with SYBR Premix Ex TaqTM II (Takara, Japan). Both of them were performed on the BioRad IQ5 system. The primer sequences (Invitrogen, USA) are shown in Table 2. The relative quantification of the target gene was normalised to U6, and calculated using the 2–△△Ct method. Melting curve profiles were produced at the end of each PCR to confirm the specific transcriptions of amplification. All samples were measured in triplicate.

**Table 2:** Primer sequences used for qRT-PCR

*U6*	F (5′CTCGCTTCGGCAGCACA)
	R (5′AACGCTTCACGAATTTGCGT)
*hCOL10A1*	F (5′CAAGGCACCATCTCCAGGAA)
	R (5′AAAGGGTATTTGTGGCAGCATATT)
*hMMP13*	F (5′GCCAAATTATGGAGGAGATGC)
	R (5′GCCGGTGTAGGTGTAGATAGGAA)
*hCEBPβ*	F (5′TGGAGACGCAGCACAAGGTCC)
	R (5′GCTTGAACAAGTTCCGCAGGGTG)
*hADAMTS5*	F (5′CAAGTGCGGAGTATGTGGAGG)
	R (5′GGTCTTTGGCTTTGAACTGTCG)
*hACAN*	F (5′CCGCTACGACGCCATCTGCTA)
	R (5′ATCACGCTGCCTCGGGCTTCAC)
*hsa-miR-193b-3p*	F (5′TGGCCCTCAAAGTCCCGCTAA)
*hsa-miR-455-3p*	F (5′GCAGTCCATGGGCATATACAC)
*hsa-miR-92a-3p*	F (5′TATTGCACTTGTCCCGGCCTGT)
*hsa-miR-381-3p*	F (5′GGGCAAGCTCTCTGTAAAAAA)

### Histology, immunohistochemistry, and in situ hybridisation

Each of the frozen tissue specimens was fixed in 10% buffered formalin, thawed, dehydrated in graded ethanol, cleared in xylene, and paraffin-embedded with their anatomical orientations preserved. Sections were cut at a thickness of 5 μm from the paraffin blocks using a microtome, mounted on saline-coated slides and stored at room temperature. All sections of the torn meniscal samples were cut from the site of the tear. The sections were stained with haematoxylin and eosin (H&E) for general cell identification. The sections were observed and photographed with a light microscope (Leica, Germany).

Immunohistochemical analysis was performed as described previously [25]. Briefly, the meniscal tissue sections were blocked in phosphate-buffered saline (PBS) plus 0.025% Tween 20 with 10% FBS, followed by incubation with diluted ADAMTS5, CEBPβ, and MMP13 protein antibodies (Abcam, UK) (diluted 1:100 in PBS) at 4 °C overnight. Negative controls were prepared using PBS instead of the primary antibody. After incubation with primary antibody, samples were probed with secondary antibody (biotinylated mouse/anti-rabbit IgG; Dako, Denmark) for 30 min at room temperature. Probes for human *miR-455-3p* and *miR-381-3p* (Exiqon, Denmark) were used. In situ hybridisation for identifying microRNA expression, was performed as previously reported [24].

### Histopathological evaluation and clinical follow-up

Based on the histology, the degeneration grade of the meniscal specimens was assessed using modified Pauli’s microscopic grading system (Table 3), which was validated to evaluate changes in three aspects: the surface of the inner border, cellularity, and collagen organisation. The range of possible total scores was 0–9, which was further categorized into 4 grades: G1 = 0–1, G2 = 2–4, G3 = 5–7, and G4 = 8–9. Grade 1 represents normal tissue, Grade 2 is mild degeneration, Grade 3 is moderate degeneration, and Grade 4 is severe degeneration. All images were captured using light microscopy (Leica, Germany). Three pathologists, who were blind to the sample grouping, evaluated the resulting slides simultaneously, and a consensus was reached. At least three sections were graded for each sample.

**Table 3:** Criteria and scores of modified Pauli’s microscopic grading system within meniscus

	Score
I Surface of inner border	
A. Smooth	0
B. Slight fibrillation or slightly undulating	1
C. Moderate fibrillation or markedly undulating	2
D. Severe fibrillation or disruption	3
II Cellularity	
A. Normal	0
B. Diffuse hypercellularity or hypocellularity	1
C. Vacuoloid changes or cell clusters	2
D. Vacuoloid changes and cell clusters	3
III Collagen organisation/alignment and fibre organisation	
A. Collagen fibres organised and homogenous eosinophilic staining of extracellular matrix	0
B. Fewer collagen fibres unorganised or diffuse foci of hyaline or mucinous degeneration	1
C. Most collagen fibres unorganised or confluent foci or bands of hyaline or mucinous degeneration.	2
D. Collagen fibres unorganised and fibrocartilaginous separation (oedema, cyst formation).	3

The range of possible total scores was 0–9, which was then converted into 4 grades: *G1* 0–1, *G2* 2–4, *G3* 5–7, and *G4* 8–9. Grade 1 represents normal tissue, Grade 2 is mild degeneration, Grade 3 is moderate degeneration, and Grade 4 is severe degeneration

Specific reactivity (IR) and the proportion of positive cells based on immunohistochemistry and in situ hybridisation were assessed. The intensity of the reaction (intensity score or IR) was stratified into four categories: 0, no IR; 1, weak IR; 2, moderate IR; and 3, strong IR. The proportion of positive cells (extent score) was scored as a percentage of the final number of 100 cells in five categories: 0, < 5% of positive cells exhibiting IR; 0.3, 5–30% of the positive cells exhibiting IR; 0.5, 31–50% of the positive cells exhibiting IR; 0.75, 51–75% of the positive cells exhibiting IR; and 1, 76–100% of the positive cells exhibiting IR. Counting was performed at 40× magnification. The product of these two values was used for calculation of the overall IR score (total score), as described by Vandeputte and Musumeci [10, 26]. All images were captured under a light microscope (Leica, Germany). Three pathologists, who were blind to the sample grouping examined all sections independently, and at least three sections were graded in each sample.

To assess the clinical outcomes, we asked all the patients undergoing meniscectomy to complete a questionnaire consisting of the Lysholm Knee Scoring Scale and International Knee Documentation Committee Subjective Knee Evaluation Form (IKDC) preoperatively and postoperatively, respectively. The mean duration of postoperative follow-up was 18.1 months (range, 15–20 months).

### Statistical analysis

Data were analysed for evaluating the statistical differences between groups using the non-parametric Kruskal-Wallis test followed by the Mann-Whitney U test. Student’s unpaired t-tests were used to compare postoperative scores between the two groups. Paired t-tests were applied to examine the differences between preoperative and postoperative scores. Statistical significance was set at *p* < 0.05. The values presented in the text, figures, and tables are given as the mean and the standard error of the mean (mean ± SEM). All statistical analyses were performed using SPSS (Version 13.0, SPSS Inc., USA).

## Results

### Morphologic and histologic observation

**Fig. 1:**
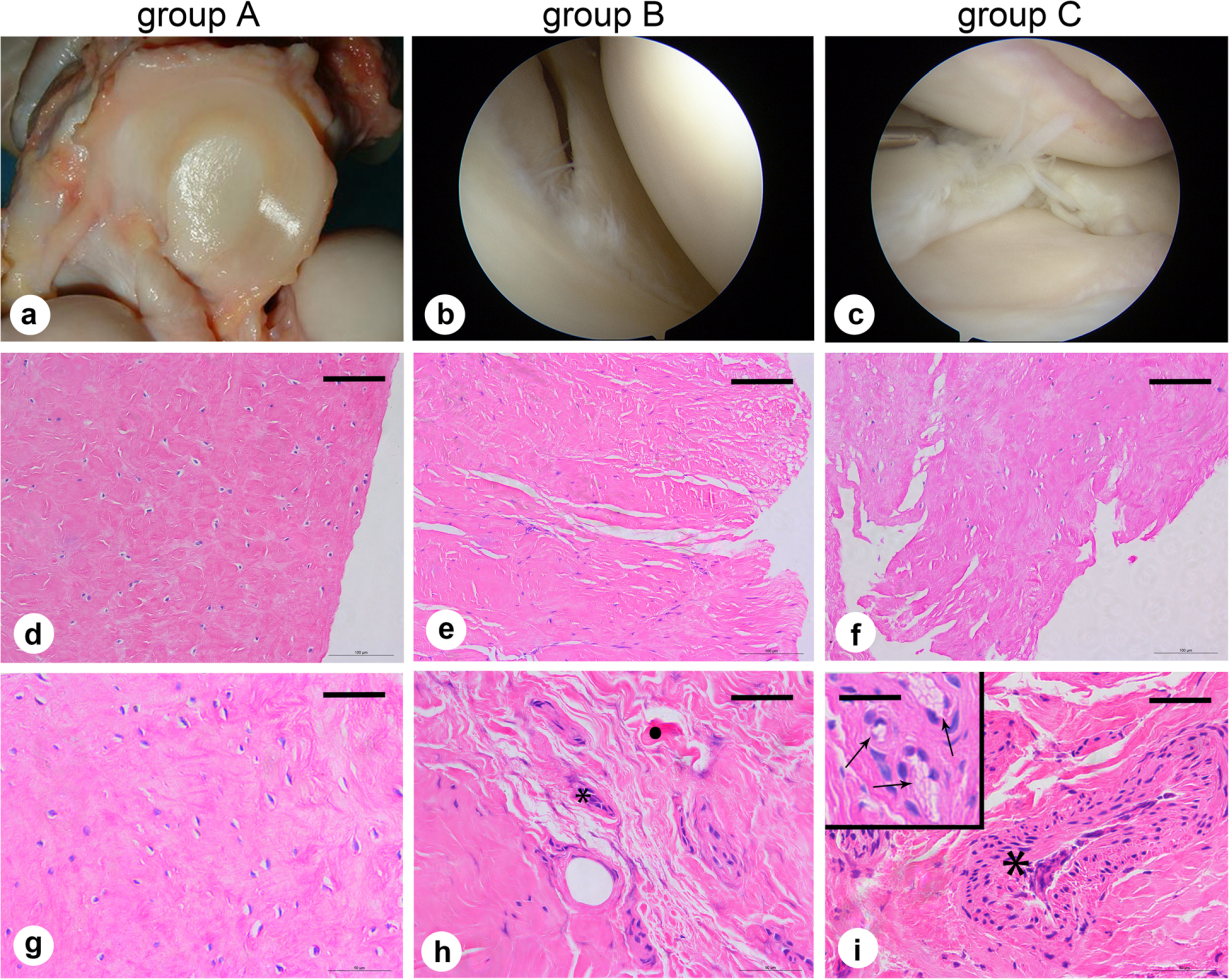
Morphological and histological observations of the meniscus. **a** Gross observation in group A (normal meniscus). **b**,**c** Arthroscopic observation in group B (simple meniscal tear) and group C (meniscal tear with concomitant ACL tear). **d**-**f** Haematoxylin-eosin staining observation (× 20); scale bars: 100 μm. **g**-**i** Haematoxylin-eosin staining observation (× 40); scale bars: 50 μm (insert: 25 μm). Vacuoloid changes are marked with arrows; cell clusters are marked with (*); hyaline degeneration are marked with (**.**). Representative results from at least three samples in each group are shown

To investigate whether there were degenerative pathological changes in the meniscus, we used modified Pauli’s microscopic grading system of the meniscus to evaluate the different groups (Table 3). In the control group (group A), general observations showed that the normal meniscus was a bright white or beige crescent-shaped tissue with intact surface (Fig. 1a). The results of H&E staining demonstrated that the surface of the meniscus was smooth with organised collagen fibres arranged tightly with homogenous eosinophilic staining, and that the round or fusiform fibrochondrocytes in the lacunae were regularly shaped with large nuclei (Fig. 1d, g).

However, there were cracks and structural alterations in the meniscus of the tissues from the experimental groups (group B and group C). Arthroscopic observation showed that the torn meniscus was incomplete with a roughly fractured pale yellow surface compared with the normal meniscus with some broken fibres floating in the operative field (Fig. 1b, c). H&E staining results showed an uneven meniscal surface that was interrupted and loose, accompanied by unorganised collagen fibres and hyaline degeneration, and the fibrochondrocytes were irregularly arranged with diffuse hypercellularity or hypocellularity in number. Localised vacuoloid changes and cell clusters were also observed (Fig. 1e, f, h, and i). The meniscal degeneration score showed that all the meniscal samples in group A were normal, the torn menisci showed mild (4 patients) and moderate (3 patients) degeneration in group B, and the meniscal samples in group C showed mild (2 patients), moderate (4 patients) and severe (1 patient) degeneration (Table 4).

**Table 4:** Modified Pauli’s meniscal degeneration score (surface of inner border, cellularity, collagen organisation) and total score

Group	Patient#	Surface	Cellularity	Collagen	Total score (grade)
Group A	#1	0	0	0	0 (normal)
	#2	0	0	0	0 (normal)
	#3	1	0	0	1 (normal)
	#4	0	0	0	0 (normal)
	#5	0	0	0	0 (normal)
	#6	0	0	0	0 (normal)
Group B	#1	3	0	1	4 (mild)
	#2	3	2	2	7 (moderate)
	#3	3	0	1	4 (mild)
	#4	3	0	1	4 (mild)
	#5	3	1	2	6 (moderate)
	#6	3	1	1	5 (moderate)
	#7	3	0	1	4 (mild)
Group C	#1	3	2	1	6 (moderate)
	#2	3	0	1	4 (mild)
	#3	3	3	2	8 (severe)
	#4	3	2	2	7(moderate)
	#5	3	0	1	4 (mild)
	#6	3	1	2	6 (moderate)
	#7	3	1	1	5 (moderate)

Group A: Normal meniscus; group B: Simple meniscal tear; group C: Meniscal tear with concomitant anterior cruciate ligament tear. Total score: G1, 0–1 normal; G2, 2–4 mild; G3, 5–7 moderate; G4, 8–9 severe

### Correlation of gene expression levels with cartilage degeneration and miRNA levels

**Fig. 2:**
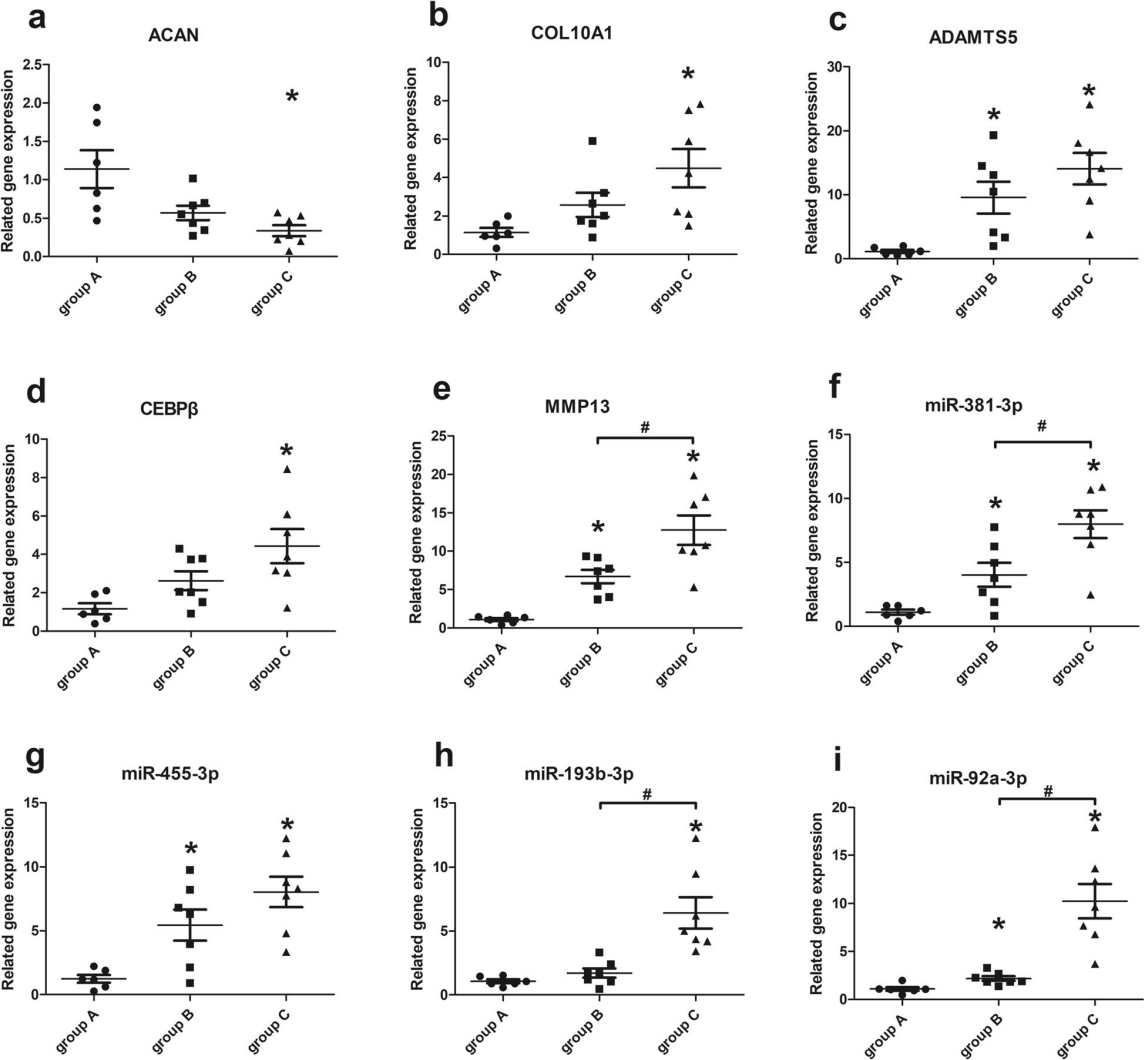
Expression levels of genes relative to cartilage degeneration and miRNA levels. Expression levels were determined using quantitative real-time polymerase chain reaction with U6 as the reference. Values are given as the mean and the standard error of mean. Within a given group, significant difference (*p* < 0.05) compared with group **a** (normal meniscus) is denoted with (*). Significant differences (*p* < 0.05) between group **b** (simple meniscal tear) and group **c** (meniscal tear with concomitant ACL tear) are marked with (#)

When compared with normal meniscal tissues without tears (group A), the expression of *ADAMTS5* (*p* = 0.001)*,* and *MMP13* (*p* = 0.001) was higher in specimens with simple meniscal tears (group B). The expression of *ADAMTS5* (*p* = 0.001)*, COL10A1* (*p* = 0.005)*, CEBPβ* (*p* = 0.005)*,* and *MMP13* (*p* = 0.001) was increased while that of *ACAN* (*p* = 0.008) was decreased in specimens with meniscal and concomitant ACL tears (group C). In addition, expression of *MMP13* (*p* = 0.011) was higher in group C than that in group B. (Fig. 2a-e).

Similarly, the levels of the miRNAs, *miR-381-3p* (*p* = 0.022), *miR-455-3p* (*p* = 0.022)*,* and *miR-92a-3p* (*p* = 0.014) were increased in the tissues from group B compared with those from group A. Similarly, when compared with group A, the levels of *miR-381-3p* (*p* = 0.001), *miR-455-3p* (*p* = 0.001)*, miR-193b-3p* (*p* = 0.001)*,* and *miR-92a-3p* (*p* = 0.001) were significantly higher in the tissues from group C. In addition, the levels of *miR-381-3p* (*p* = 0.017), *miR-193b-3p* (*p* = 0.001)*,* and *miR-92a-3p* (*p* = 0.001) were significantly higher in group C compared with those in group B. (Fig. 2f-i).

### Immunohistochemistry and in situ hybridisation

**Fig. 3:**
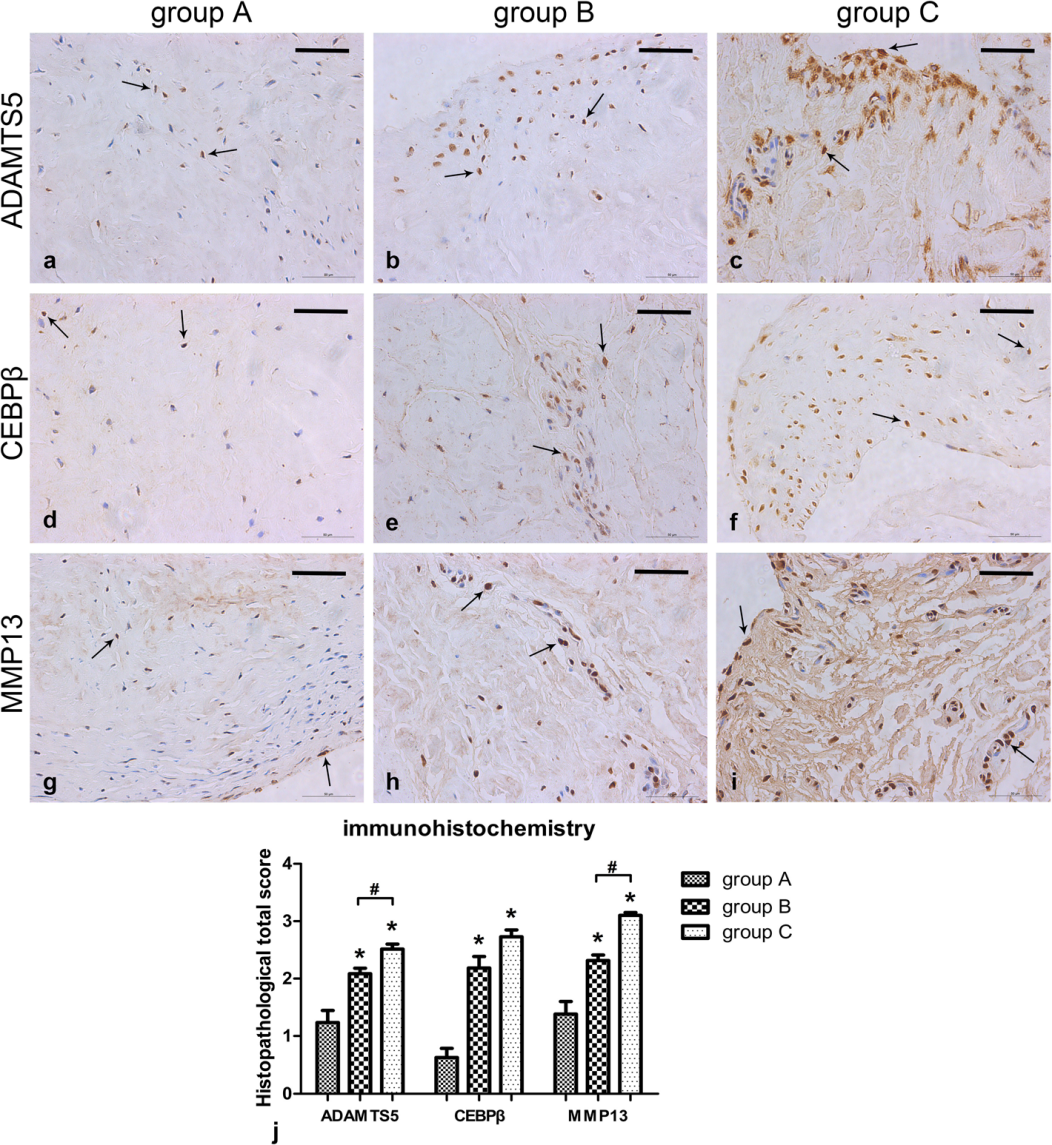
Immunohistochemical results showing ADAMTS5, CEBPβ, and MMP13 levels in the meniscus. **a**-**i** Sections were counterstained with haematoxylin, representative immunohistochemical positive cells are marked with arrows, and the scale for the bar is 50 μm. **j** Immunohistochemistry graph showing total histopathological score in group A (normal meniscus), group B (simple meniscal tear), and group C (meniscal tear with concomitant ACL tear), taken as the product of the specific reactivity (IR) and proportion of positive cells. The values are given as the mean and the standard error of mean. Within a given group, significant difference (*p* < 0.05) compared with group A is denoted with (*). Significant differences (*p* < 0.05) between group B and group C are marked with (#). Representative results from at least three samples in each group are shown

To validate the mRNA qRT-PCR results, we conducted immunohistochemistry with specific antibodies to detect three representative cartilage degenerative related factors, including ADAMTS5, CEBPβ, and MMP13. Positive immunohistochemical staining was defined as the presence of brown chromogen. The results showed that in the three different groups, positive staining for ADAMTS5 and MMP13 in the explanted tissue was observed in the cells or in the pericellular space, while that for CEBPβ was primarily on the edge of the haematoxylin-stained cell nucleus and distributed within the cytoplasm or in the immediate lacuna (Fig. 3a-i). Moreover, compared to fibrochondrocytes in group A, the staining for ADAMTS5 (*p* = 0.014), CEBPβ (*p* = 0.002), and MMP13 (*p* = 0.005) was higher in fibrochondrocytes of group B, whlie ADAMTS5 (*p* = 0.001), CEBPβ (*p* = 0.001), and MMP13 (*p* = 0.001) staining was highest in fibrochondrocytes of group C. Furthermore, fibrochondrocytes in group C strongly expressed ADAMTS5 (*p* = 0.011) and MMP13 (*p* = 0.001) than those in group B (Fig. 3j).

**Fig. 4:**
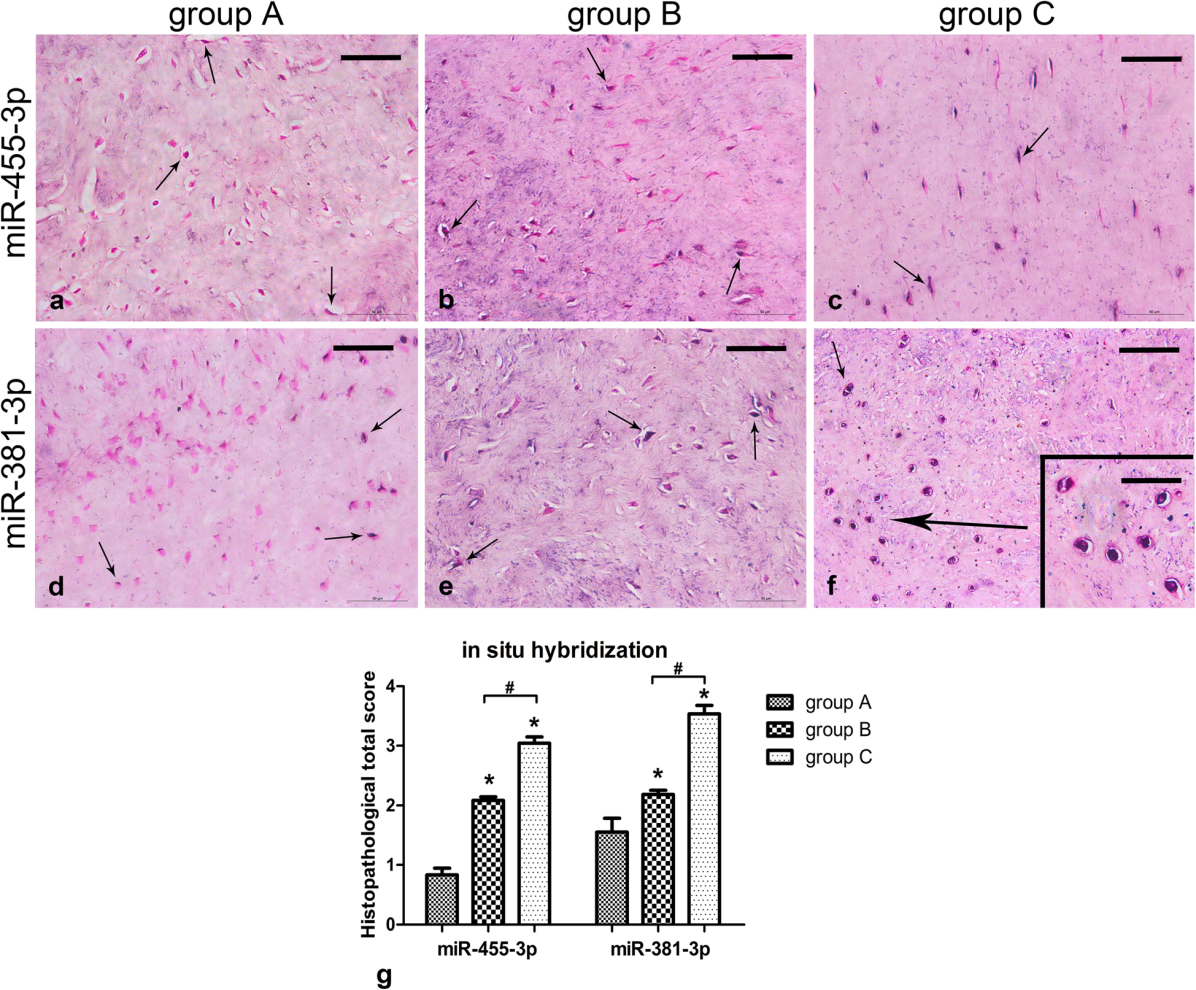
In situ hybridisation results showing *miR-455-3p* and *miR-381-3p* levels in the meniscus. **a**-**f** Sections were counterstained with nuclear fast red solution, representative positive cells are marked with arrows, and the scale for the bar is 50 μm (insert: 25 μm). **g** In situ hybridisation graph showing total histopathological score in group A (normal meniscus), group B (simple meniscal tear),and group C (meniscal tear with concomitant ACL tear), taken as the product of the specific reactivity (IR) and proportion of positive cells. The values are given as the mean and the standard error of mean. Within a given group, significant difference (*p* < 0.05) compared with group A is denoted with (*). Significant differences (*p* < 0.05) between group B and group C are marked with (#). Representative results from at least three samples in each group are shown

To verify the results of miRNA qRT-PCR, *miR-455-3p* and *miR-381-3p* were detected via in situ hybridisation. Positive cells were clearly displayed with prominent localisation of the miRNAs mainly in the nucleus (Fig. 4a-f). When compared with group A, the total histopathological scores of *miR-455-3p* (*p* = 0.001) and *miR-381-3p* (*p* = 0.035) in fibrochondrocytes were higher in group B, *miR-455-3p* (*p* = 0.001) and *miR-381-3p* (*p* = 0.001) were significantly higher in group C. In addition, the total scores of *miR-455-3p* (*p* = 0.001) and *miR-381-3p* (*p* = 0.035) were higher in group C than those in group B (Fig. 4g). All detected factors and miRNAs relative to cartilage degeneration were differentially expressed in the meniscus among the different groups, with substantial consistency in the qRT-PCR results.

### Clinical follow-up

**Fig. 5:**
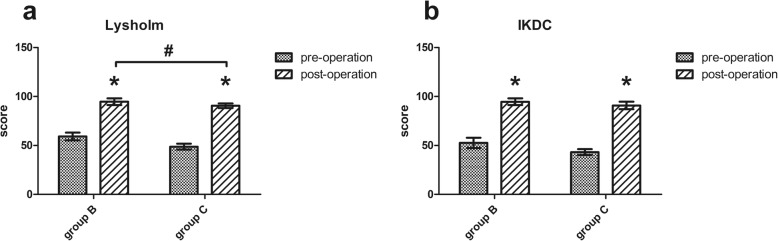
Lysholm Knee Score scale and IKDC during the period of pre-operation and post-operation. The values are given as the mean and the standard error of mean. Within a given group, significant difference (*p* < 0.05) compared with preoperative score is denoted with (*). Significant differences (*p* < 0.05) in postoperative score between group B (simple meniscal tear) and group C (meniscal tear with concomitant ACL tear) are marked with (#)

To evaluate the effects of the surgery and knee function during early postoperative period, we did a follow-up on the patients who underwent meniscectomy (group B and group C). Postoperative Lysholm scores were significantly higher than the preoperative scores for both group B (*p* = 0.000) and group C (*p* = 0.000). Similarly, postoperative IKDC scores in both group B (*p* = 0.000) and group C (*p* = 0.000) were significantly higher than the preoperative scores. These results indicate that the effect of arthroscopic surgery was satisfactory in all patients. Besides, postoperative Lysholm score was higher in group B than that in group C (*p* = 0.020) (Fig. 5).

## Discussion

Pauli C et al. used Pauli’s microscopic grading system to validate the changes observed in three separate areas (femoral side, tibial side, and inner border) of ageing and osteoarthritic (OA) menisci [27]. In our study, the meniscal biopsies were taken only from the inner border and all the sample sections were cut from the site of the tear. Additionally, we focused on torn meniscus in this study, which is different from ageing and OA menisci based on in Pauli’s research. Therefore, we modified the Pauli’s grading system to make it more suitable for our research (Table 3). In our results, meniscal biopsies collected from the patients with meniscal tear (group B and group C) showed evidence of hyaline degeneration and loss of collagen fibre organisation. Although the features from the meniscal samples in both the groups were very different from those obtained from the amputee controls (group A), they were similar to those in the OA patients, as described by Pauli et al. [27]. Vacuoloid changes and cell cluster formation in the meniscus may occur as a consequence of alterations in the tissue structure during OA development [28–31]. We found that the torn menisci in groups B and C exhibited vacuoloid changes or/and abnormal cell clusters. Consistent with our findings, Battistelli M et al. found vacuoloid changes in the menisci from traumatic and end-stage OA patients [32]. However, contrary to Battistelli’s observation, we evaluated the score for the surface of the inner border in groups B and C to be 3 (Table 4), as we found that all torn menisci showed severe fraying and disruption on the surface. Microscopically, we found that there were varying degrees of degenerative pathological changes in the torn menisci versus normal menisci, thus providing new insights into the pathological changes in the meniscus after injury, especially when combined with a torn ACL.

In our research, a 34-year-old female patient in group C with a certain type of complex meniscal tear (flap and horizontal tear) in the posterior horn, who had undergone arthroscopic surgery 16 months after injury, showed vacuoloid changes, cell clusters, mostly unorganised collagen fibres and some hyaline degeneration. She was the only patient with severe meniscal degeneration (score of 8). Several studies demonstrated that age, gender, BMI, sports activities and time interval from injury to surgery influenced the risk of meniscal degeneration [8, 33]. Consequently, the combined injury pattern (meniscal tear and ACL tear) might not be the only factor contributing to meniscus degeneration in this case and other factors, such as higher age, feminine gender, longer interval between injury and arthroscopy, might also increase the risk of meniscal degradation as well. We will attempt to address these assumptions in our future studies.

It is considered that meniscus degeneration, similar to cartilage degradation, is due to metabolic imbalances and is characterised by increased synthesis and activity of matrix metalloproteinases (MMPs) and aggrecanases [11, 34]. Several studies have demonstrated that both MMP13 and ADAMTS5 are master ECM degenerative enzymes that have been previously considered to be major contributors to the development of joint degeneration [35, 36]. Brophy’ findings suggested that the expression level of *MMP13* was higher in patients with a combined meniscal and ACL tear compared with the patients with only meniscal tear. However, there was no significant difference in the *ADAMTS5* expression in both groups [8]. Similarly, our results demonstrated that in the presence of meniscal tear, the expression of *MMP13* and *ADAMTS5* is elevated, especially when it is associated with ACL injury, but without any significant difference in the *ADAMTS5* levels between the groups B and C. Moreover, these results were consistent with the immunohistochemical staining results. The overexpression of matrix-degrading genes is likely to be responsible for the degradation of both aggrecan and collagen. Besides, it is implied that the catabolic processes might have occurred in the meniscus after it was torn, and when combined with ACL injury, these substantial changes appear to become significant. Furthermore, the ECM degenerative enzymes that are produced in the meniscus probably not only act on the meniscus itself but can also be released into the joint cavity to degrade the cartilage, which could play a critical role in the subsequent articular degradation.

Conversely, when compared with patients with a normal meniscus, the expression of *ACAN* in patients with meniscal tear was decreased. Additionally, *ACAN* was expressed at dramatically lower levels in patients with a combined meniscal and ACL rupture. These findings indicate that the anabolic ability decreases in the lacerated meniscus, which could explain why the torn meniscus has less potential for repair, particularly when combined with ACL injury. In addition, the metabolism of the meniscal tissues resected during surgery may provide vital insights into the condition of the integral articular health, which is probably an important indicator for predicting future degradation and the latent risk for subsequent development of OA [37]. However, contrary to the “significantly lower” values shown in Brophy’ results [8], we found the levels of *ACAN* were not significantly different between groups B and C. We speculate that this discrepancy might be due to the differences in the patients’ age as all the patients we recruited were under 40, which is in contrast with some of the patients in Brophy’ study who were 60 years old. Age might be a factor in influencing the gene expression in meniscal tissue.

Type X collagen (COL10A1) is a short, network-forming collagen specifically expressed by hypertrophic chondrocytes and is regarded as an important hypertrophic marker of chondrocytes [38, 39]. Behrendt reported that TNF-α increased the expression of *COL10A1* in the meniscus of explants [38, 39]. Brophy suggested that *COL10A1* was the most prominent mRNA to be elevated in OA meniscus compared to that in the injured meniscus [9]. Similarly, our findings showed that meniscal tears result in elevated expression of the *COL10A1* in the meniscal tissues compared with the normal meniscus. In addition, combined meniscal and ACL damage has a higher tendency to get exacerbated than insular meniscal damage. Furthermore, vacuoloid changes and cell clusters of fibrochondrocytes with reduced lacuna in the torn meniscus can be clearly seen via microscopic observation, suggesting that the phenotype of some fibrochondrocytes in the torn meniscus switch from normal to hypertrophic. It is well known that chondrocyte hypertrophy contributes to cartilage degeneration during the progression of OA [40]. Therefore, this may be another piece of evidence to support that in the long-run, the meniscus tends to degenerate after meniscal tear, especially when accompanied by ACL rupture.

Recent research showed that CEBPβ, a transcription factor, is essential for the degeneration of cartilage in OA, which mediates the promotion of catabolic activities involving the up-regulation of MMP3, MMP13, and ADAMTS5 [16, 17]. CEBPβ is also an important regulator in facilitating the transition of chondrocytes from proliferative to hypertrophic form, which expresses type X collagen [41]. Consequently, higher levels of *CEBPβ* in patients with a meniscal tear, particularly in those with meniscal and ACL tears, might be especially correlated to higher expression of the downstream genes relative to cartilage degeneration noted earlier, which ultimately lead to elevated degradation of ECM. In other words, our observations support the possibility that the up-regulation of *CEBPβ* can explain the overexpression of *MMP13*, *ADAMTS5*, and *COL10A1*. Yet, surprisingly, there is still a lack of compelling evidence in support of this hypothesis, and this warrants further investigation.

MicroRNAs (miRNAs), small non-coding single-stranded RNAs, play an increasingly crucial role in OA progression [18, 42]. Some authors have suggested that *miR-193b-3p* affects chondrocyte ageing by regulating aggrecan, type-II collagen, and SOX9 [43]. Likewise, Tracey suggested that *miR-455-3p* exacerbates the process of OA by regulating TGF signalling and suppressing the Smad2/3 pathway [44]. Taking the aforementioned points into account, we initially inferred that the four miRNAs were probably involved in the inflammatory responses of meniscus destruction. One attractive possibility is that they regulate the target upstream signalling pathways or specific transcription factors, such as CEBPβ, NFKBIA, Runx2, SOX5, SOX9, MAPK1, SMAD3, and BMPR2 [45], respectively or synergistically, which gives rise to aberrant expression of multiple catabolic and anabolic genes. However, the exact processes used by the four miRNAs were unclear. Therefore, further investigations, such as cell culture, animal experiment and luciferase assay, are required.

It is known that both the IKDC and Lysholm scales are perceived as valid, reliable, and responsive self-reported outcome measures [46]. Our short-term results showed that the postoperative scores were significantly higher than the preoperative ones, suggesting that the clinical efficacy of arthroscopic surgery ranges from good to excellent. To minimise the effects caused by differences in the follow-up duration, we selected a minimum postoperative period of 15 months as the follow-up time, as we believe that after arthroscopy, normal knee function recovers to a relatively stable level in the patients within this duration. Nevertheless, regardless of Lysholm or IKDC scores, individuals with combined meniscal and ACL injuries showed less favourable postoperative outcomes than those with isolated meniscal tears, indicating that the knee with the associated rupture has lower functional rehabilitation and athletic ability. It was perplexing that there were significant differences in the Lysholm score while the IKDC score did not change significantly between the two groups after surgery. However, this inconsistency can be explained by the design of both the scores. The Lysholm score focuses on the symptoms and daily activities, while the IKDC score highlights the sports-related functions. Moreover, patients need more time to regain their exercise capacity after surgery.

Patients with combined injuries appear to have suffered more severe trauma and undergone a more complicated arthroscopic operation involving ACL reconstruction, which might be the most rational explanation for the lower scores. However, the discrepancy in postoperative outcomes also suggests that the joints in these patients might be more seriously damaged, which could be due to the torn meniscus not only at the biomechanics level but also at the molecular level. There was a moderately negative correlation between the MMP3:TIMP2/3 ratio in the knee synovial fluid and preoperative Lysholm score (the greater the ratio, the worse the Lysholm score) [47]. Similarly, Scanzello et al. reported that CCR7 and CCL19 expression in the biopsies of knee synovium showed strong negative associations with preoperative Lysholm scores in meniscectomy patients. Additionally, IL-8 and CCL5 were moderately but not significantly associated with Lysholm scores [48]. In the follow-up, the relative expression levels of CCL19 and CCR7 in the synovium were associated with greater postoperative improvements in the Lysholm score [49]. Consequently, there might be a potential relationship between the preoperative or postoperative Lysholm score and the molecular markers in the meniscus that were shown in our results. This provides a good starting point for discussion and further research.

This study has several limitations. For instance, the number of patients is too small. Moreover, the general health conditions including sex and smoking status should be taken into account as well. Furthermore, our clinical follow-up results lack direct evidence, such as postoperative X-ray and magnetic resonance imaging results to demonstrate that patients with combined injury patterns have more serious degeneration of meniscus and articular cartilage compared with patients with isolated meniscal tears. Here, we have compiled most of the work, which has so far only scratched the surface of this prolific field of research, and further studies need to be performed to address these issues.

## Conclusions

Our findings suggest that the torn meniscus, particularly when combined with a torn ACL, has an intrinsic tendency to get degraded at the molecular level. Additionally, *miR-381-3p, miR-455-3p, miR-193b-3p,* and *miR-92a-3p* may contribute to the progression of meniscus degeneration, which might act as potential biomarkers for subsequent development of OA. Finally, our clinical results show that patients with a combined injury pattern may have relatively worse joint function.

## Data Availability

The datasets used and/or analysed during the current study are available from the corresponding author on reasonable request.

## Abbreviations

ACAN: Aggrecan
ACL: Anterior cruciate ligament
ADAMTS5: A Disintegrin and Metalloproteinase with Thrombospondin Motifs − 5
BMI: Body mass index
BMPR2: Bone morphogenetic protein receptor 2
CEBPβ: CCAAT/enhancer binding protein beta
COL10A1: Type X collagen
COL1A1: Type I collagen
COL2A1: Type-II collagen
ECM: Extracellular matrix
FBS: Foetal bovine serum
H&E: Haematoxylin and eosin
IKDC: International Knee Documentation Committee Subjective Knee Evaluation Form
IL-8: Interleukin 8
IR: Specific reactivity
MAPK1: Mitogen activated kinase-like protein 1
miR-193b-3p: MicroRNA-193b-3p
miR-381-3p: MicroRNA-381-3p
miR-455-3p: MicroRNA-455-3p
miR-92a-3p: MicroRNA-92a-3p
miRNA: MicroRNA
MMP3, 13: Matrix metallopeptidase - 3, 13
MMPs: Matrix metalloproteinases
MRI: Magnetic resonance imaging
NFKBIA: Nuclear factor of kappa light polypeptide gene enhancer in B-cells inhibitor-alpha
OA: Osteoarthritis
PBS: Phosphate-buffered saline
qRT-PCR: Quantitative real-time polymerase chain reaction
Runx2: Runt-related transcription factor 2
SEM: Standard error of the mean
SMAD3: Mothers against decapentaplegic homolog 3
SOX5, 9: Sex determining region Y (SRY)-box 5, 9
TIMP2,3: Tissue inhibitor of metalloproteinase-2,3
TNF-α: Tumour necrosis factor-alpha
